# Angelica Sinensis Polysaccharide Suppresses Epithelial-Mesenchymal Transition and Pulmonary Fibrosis via a DANCR/AUF-1/FOXO3 Regulatory Axis

**DOI:** 10.14336/AD.2019.0512

**Published:** 2020-02-01

**Authors:** Weibin Qian, Xinrui Cai, Qiuhai Qian, Dongli Wang, Lei Zhang

**Affiliations:** ^1^Department of Lung Disease, Affiliated Hospital of Shandong University of Traditional Chinese Medicine, Jinan, Shandong 250011, China.; ^2^Department of Traditional Chinese Medicine, Shandong Academy of Occupational Health and Occupational Medicine, Shandong First Medical University & Shandong Academy of Medical Sciences, Jinan, Shandong 250062, China.; ^3^Department of Endocrinology, Affiliated Hospital of Shandong University of Traditional Chinese Medicine, Jinan, Shandong 250011, China.; ^4^Department of Personnel Section, Affiliated Hospital of Shandong University of Traditional Chinese Medicine, Jinan, Shandong 250011, China.; ^5^Department of Cardiology, Affiliated Hospital of Shandong University of Traditional Chinese Medicine, Jinan, Shandong 250011, China

**Keywords:** idiopathic pulmonary fibrosis, angelica sinensis polysaccharide, DANCR, AUF1, FOXO3

## Abstract

Idiopathic pulmonary fibrosis (IPF) is characterized by the accumulation of lung fibroblasts and extracellular matrix deposition. Angelica sinensis polysaccharide (ASP), the major bioactive component that can extracted from roots of angelica, plays functional roles in immunomodulation, anti-tumor activity, and hematopoiesis. Emerging evidence has suggested that long noncoding RNAs (lncRNAs) play important roles in pathophysiological processes in various diseases. However, the roles of lncRNAs and ASP in IPF remain poorly understood. In the present study, we investigated the effects of ASP in IPF, as well as their functional interactions with lncRNA DANCR (differentiation antagonizing non-protein coding RNA). IPF models were established by treating Sprague-Dawley rats with BLM and treating alveolar type Ⅱ epithelial (RLE-6TN) cells with TGF-β1. Our results showed that ASP treatment suppressed pulmonary fibrosis in rats and fibrogenesis in RLE-6TN cells. The lncRNA DANCR is downregulated after ASP treatment in both rat lung tissues and RLE-6TN cells, and DANCR overexpression dramatically reversed the suppressive effects of ASP in IPF. Mechanistically, DANCR directly binds with AUF1 (AU-binding factor 1), thereby upregulating FOXO3 mRNA and protein levels. Moreover, overexpression of AUF1 or FOXO3 reversed the functional effects induced by ASP treatment. In conclusion, our findings showed that DANCR mediates ASP-induced suppression of IPF via upregulation of FOXO3 protein levels in an AUF1-dependent manner. Therefore, DANCR could serve as a promising therapeutic target in IPF treatment with ASP.

Idiopathic pulmonary fibrosis (IPF) is a progressive and usually fatal lung disease characterized by activated fibroblasts/myofibroblasts, fibroblast proliferation, and excessive deposition of extracellular matrix [1]. It is currently recognized that myofibroblasts derived from epithelial cells undergo the epithelial-mesenchymal transition (EMT) and exhibit abnormal proliferation and ECM overproduction, resulting in the development of IPF. Approximately one-third of fibroblasts have been reported to have epithelial origin in pulmonary fibrosis [2], suggesting a tight interaction between the epithelial-mesenchymal transition (EMT) and fibrosis [3]. However, the exact regulatory mechanisms underlying EMT in IPF remain unclear, and the effective diagnostic markers and therapeutic options for IPF are very limited. Therefore, elucidating the mechanisms underlying IPF, identifying promising early detection markers, and developing effective therapies are important for improving the outcome of IPF patients.

Angelica sinensis (Oliv.) Diels, a perennial herb, has been broadly used as herbal medicine in Asian countries, such as China, Japan, and Korea [4]. Angelica sinensis polysaccharide (ASP) is the major bioactive component extracted from the roots of angelica. There are about 36 polysaccharides identified from ASP. The majority of the polysaccharides reported in literature are heteropolysaccharides [5]. Recently, studies have demonstrated that ASP has various biological functions, including immunomodulation, anti-tumor activity, and hematopoiesis [6-8]. However, the potential role of ASP in IPF and the underlying molecular mechanism need to be further explored.

Long noncoding RNAs (lncRNAs) are a new type of RNAs that contain more than 200 nucleotides and are not translated into proteins. LncRNAs play critical roles in the regulation of cell growth, apoptosis, migration, invasion, drug resistance, and chromatin remodeling [9]. LncRNAs play important roles in regulating downstream target genes by modifying chromatin structure in the nucleus and acting as competing endogenous RNA (ceRNAs) in the cytoplasm [10-13]. Previous studies revealed that lncRNAs participate in various cellular processes and that the dysregulation of lncRNAs usually leads to the o development of diseases, such as cancer [14] and cardiovascular diseases [15]. Moreover, several studies indicated that certain lncRNAs, such as PFAR [16], lncITPF [17], PFRL [18] and PFAL [19], act as important regulators in the progression of IPF. However, the exact functions of lncRNAs in IPF remain elusive.

The lncRNA DANCR (differentiation antagonizing non-protein coding RNA) is a newly identified lncRNA with pivotal roles in cell proliferation, migration, invasion, and stem cell differentiation [20-22]. DANCR was first described as a lncRNA that blocks the differentiation of the epidermal progenitor cells [23, 24]. Moreover, DANCR was shown to promote EMT progression and the invasion capability of malignant cells [22, 25]. However, the functional association between DANCR and IPF is rarely reported.

TGF-β1 is a major fibrogenic factor that was shown to promote the EMT and lung fibroblast-to-myofibroblast trans-differentiation [26]. In our previous study, we established a rat model of pulmonary fibrosis by intratracheal administration of bleomycin (BLM) and showed that the IPF in rats could be dramatically reversed by suppressing EMT induced by TGF-β1 treatment [27, 28]. In the current study, we sought to identify the functional effects and the regulatory mechanisms by which ASP regulates EMT and IPF based on the established model. By performing a series of experimental analysis, we discovered that ASP inhibited proliferation, the EMT, and fibrogenesis of alveolar type Ⅱ epithelial (RLE-6TN) cells *in vitro*, as well as pulmonary fibrosis *in vivo*. Mechanistically, ASP downregulates the expression of DANCR, which in turn inhibits the interaction between DANCR and AUF1 and the translation of FOXO3 mRNA.

## MATERIALS AND METHODS

### IPF model and treatment

The IPF model was established by the intratracheal instillation of Sprague-Dawley rats (180 g-220 g) with 50 mL of saline containing BLM (5 mg/kg, Nippon Kayaku, Japan) for 14 days as previously described [27]. ASP was intragastrically administered at 20 mg/kg for another 14 days following BLM treatment. At the same time, the adenovirus-associated packaging DANCR sequence or negative control sequence was sprayed into the rat lung tissues using a PennCentury MicroSprayer (Penn-Century Inc., PA, USA). The animal studies were approved by the Ethics Committee of Affiliated Hospital of Shandong University of Traditional Chinese Medicine.

### Pathological staining

The hematoxylin and eosin (H&E) staining, Masson’s trichrome staining, and immunohistochemistry (IHC) assay were performed as previously described [27]. For H&E and Masson’s staining, the lung tissues stripped from rats were fixed with 4% paraformaldehyde for 24 h, embedded in paraffin, cut into 5-μm-thick sections, and then stained with H&E or Masson’s trichrome kit (Nanjing Jiancheng Co., Ltd., China) according to the manufacturer’s instructions. For IHC analysis of collagen1 and FOXO3 expression, tissues were incubated overnight at 4 °C with respective primary antibodies: anti-collagen-1 (1:500, Abcam, cat. no. ab34710) or anti-FOXO3 (1:500, Abcam, cat. no. ab17026). The detection system used was 3,30-diaminobenzidine (DAB) from DAKO (Santa Clara, CA). Slides were counterstained using hematoxylin.

### Cell culture and treatment

Alveolar type Ⅱ epithelial (RLE-6TN) cells were obtained from the American Type Culture Collection (Manassas, VA, USA). Cells were cultured in Ham’s F12 medium (10% fetal bovine serum, FBS) at 37 ºC and 5% CO_2_. The EMT cell model was generated by exposing RLE-6TN cells in Ham’s F12 medium (10% FBS) containing 10 ng/ml TGF-β1 (Promega, Madison, WI) for 48 h as previously described [28]. ASP (98% purity) was purchased from Yongye Bio-engineering Co., Ltd. (Shanghai, China) and used at 200 μg/mL for 12 h.

### Vector construction and cell transduction

The silencing RNA targeting AUF1 (si-AUF1) and FOXO3 (si-FOXO3) were purchased from Ribo Bio (Guangzhou, China). Negative control siRNA was purchased from Invitrogen (CAT#12935-110, Carlsbad, CA, USA). The full-length DANCR overexpression plasmid (p-DANCR) was purchased from GeneChem (Shanghai, China) and cloned into adenoviruses. The adenovirus-associated packaging DANCR sequence or negative control sequence was sprayed into the rat lung tissues using a PennCentury MicroSprayer (Penn-Century Inc., PA, USA). Cell lines were transfected using Lipofectamine 2000 (Life Technologies, USA) for 6 h at the final concentration of 100 nM according to the manufacturer’s instructions. In our study, cells may be treated with ASP and transfected simultaneously. The sequences of the small interfering RNAs used in the study are presented in Table 1.

**Table 1:** Information of the qPCR primer sequences and silencing RNA sequences.

qPCR primer name	Sequence (5’-3’)
DANCR (Forward)	TCGGAGGTGGATTCTGTTAGG
DANCR (Reverse)	TCGGTGTAGCAAGTCTGGTGA
FOXO3 (Forward)	CTCCACCCCTGCTGAGATGAT
FOXO3 (Reverse)	AGTGAGAACGTTGTCCCGCGCTGG
GAPDH (Forward)	GCACCGTCAAGGCTGAGAAC
GAPDH (Reverse)	ATGGTGGTGAAGACGCCAGT
U6 (Forward)	CTCGCTTCGGCAGCACA
U6 (Reverse)	AACGCTTCACGAATTTGCGT
U1 (Forward)	GGGAGATACCATGATCACGAAGGT
U1 (Reverse) Silencing RNA name	CCACAAATTATGCAGTCGAGTTTCCC Sequence (5’-3’)
si-AUF1 si-FOXO3 si-NC	UCGACUAUCUGCUCCAAG TGATCAGCCTCAATCTGCA GACCTACAACTACCTATCA

### Reverse transcription (RT) and quantitative real-time polymerase chain reaction (qRT-PCR)

The lncRNA expression levels were measured by qRT-PCR using SYBR Premix Ex Taq^TM^ kit (Takara) and a StepOnePlus Real-Time PCR System (Applied Biosystems, Carlsbad, CA, USA). The 2^-ΔΔCT^ values relative to one of the samples was calculated to analyze relative expression levels. GAPDH was used to normalize the relative expression levels of the lncRNAs and mRNAs. The relative expression levels of different were was calculated using the ΔΔCT method [29]. The primer sequences are presented in Table 1.

### Cell proliferation assay

The proliferation of transfected cells after ASP treatment was evaluated by Cell Counting Kit-8 (Dojindo Molecular Technologies, Inc., Kumamoto, Japan) following the manufacturer’s protocol. Cells were seeded at a density of 3,000 cells/well in 96-well culture plates and cultured for 18 h prior to transfection. After transfection or (and) ASP treatment for 48 h, the culture medium was added with 10% CCK-8 solution. Three independent measurements were performed. The optical density was measured at the wavelength of 450 nm using Thermo Scientific Multiskan FC at 2 days post-transfection. The cell proliferation rate was calculated based on the number of viable cells.

### Cell migration assay

Cell migration and invasion are determined by Transwell assays. Cells cultured in serum-free Ham’s F12 medium were added onto each well of the chambers without Matrigel. Ham’s F12 with 10% FBS was added to the lower chamber. After incubation for 24 h, cells in the upper chamber were removed, whereas the cells on the lower membrane surface were fixed and stained with 0.5% crystal violet. Cells from at least five random fields of each well were counted under a microscope (Olympus).

### RNA fluorescence in situ hybridization (RNA FISH)

Cy3-labeled DANCR probes were obtained from RiboBio (Guangzhou, China). Hybridizations were carried out using Fluorescence in Situ Hybridization Kit (RiboBio, China) according to the manufacturer's instructions. Briefly, 4% paraformaldehyde was used to fix the alveolar epithelial cells, followed by treatment with 0.5% Triton. Then, cells were incubated with the specific probes overnight. All fluorescence images were captured using Nikon A1Si Laser Scanning Confocal Microscope (Nikon Instruments Inc, Japan).

### RNA pulldown

Briefly, about 1×10^7^ RLE-6TN cells were harvested, crosslinked, lysed, and sonicated. The cell lysates were incubated with appropriate DANCR probe or oligo probe at 37 °C for 4 h in Hybridization Buffer. Subsequently, lysates with or without DANCR-probe complex were incubated with appropriate C-1 magnetic beads (Life Technologies) for 30 min. Then, the beads were thoroughly washed with wash buffer, and the proteins remaining on the beads were separated by western blotting.

### RIP assay

RIP experiments were performed using a Magna RIP™ RNA-Binding Protein Immunoprecipitation Kit (Millipore, USA) following the manufacturer's instructions. The anti-AUF1 (ab61193, Abcam) antibody was used in the RIP assays. Normal IgG was used as negative control for immunoprecipitation, and GAPDH was used as the internal control for qRT-PCR.

**Figure 1.**
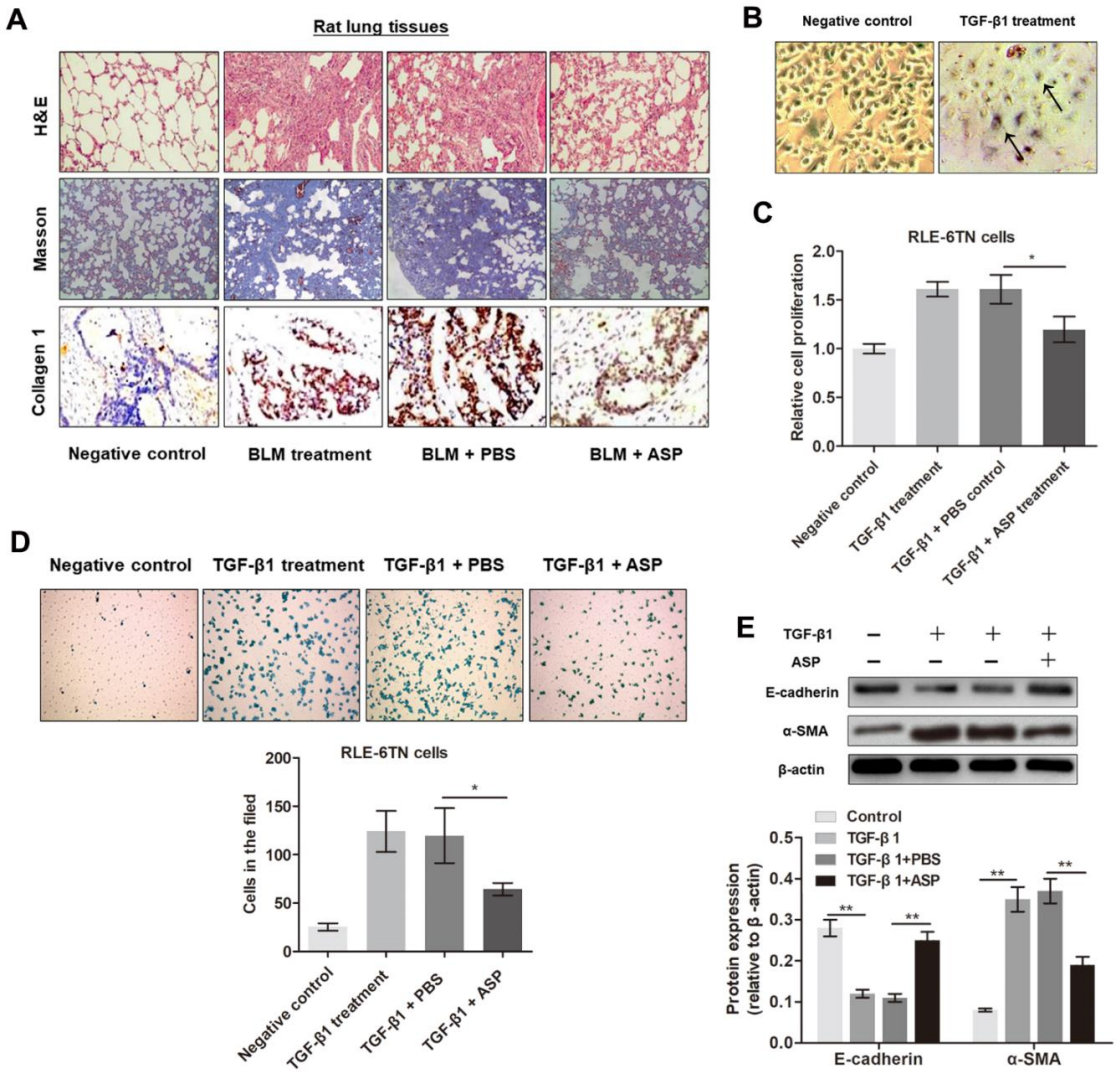
**ASP treatment inhibits pulmonary fibrosis *in vivo* and cell fibrogenesis *in vitro***. **(A)** H&E and Masson’s staining assays were performed to demonstrate the effects of BLM and ASP on pulmonary fibrosis of rat lung tissues. Immunohistochemical analysis of collagen-1 protein levels in rat lung tissues. **(B)** Morphological changes in RLE-6TN cells, such as disappearance of intercellular junction and spindle-like structures (indicated by arrows), were observed after treatment with TGF-β1. **(C)** Cell proliferation was evaluated by performing CCK8 assay in the different sample groups, ^*^P<0.05. **(D)** Transwell assay was performed to detect the migration of RLE-6TN cells, ^*^P<0.05. **(E)** The expression levels of E-cadherin and α-SMA in RLE-6TN cells were measured by western blotting. Protein expression levels relative to β-actin levels are shown, ^**^P<0.01.

**Figure 2.**
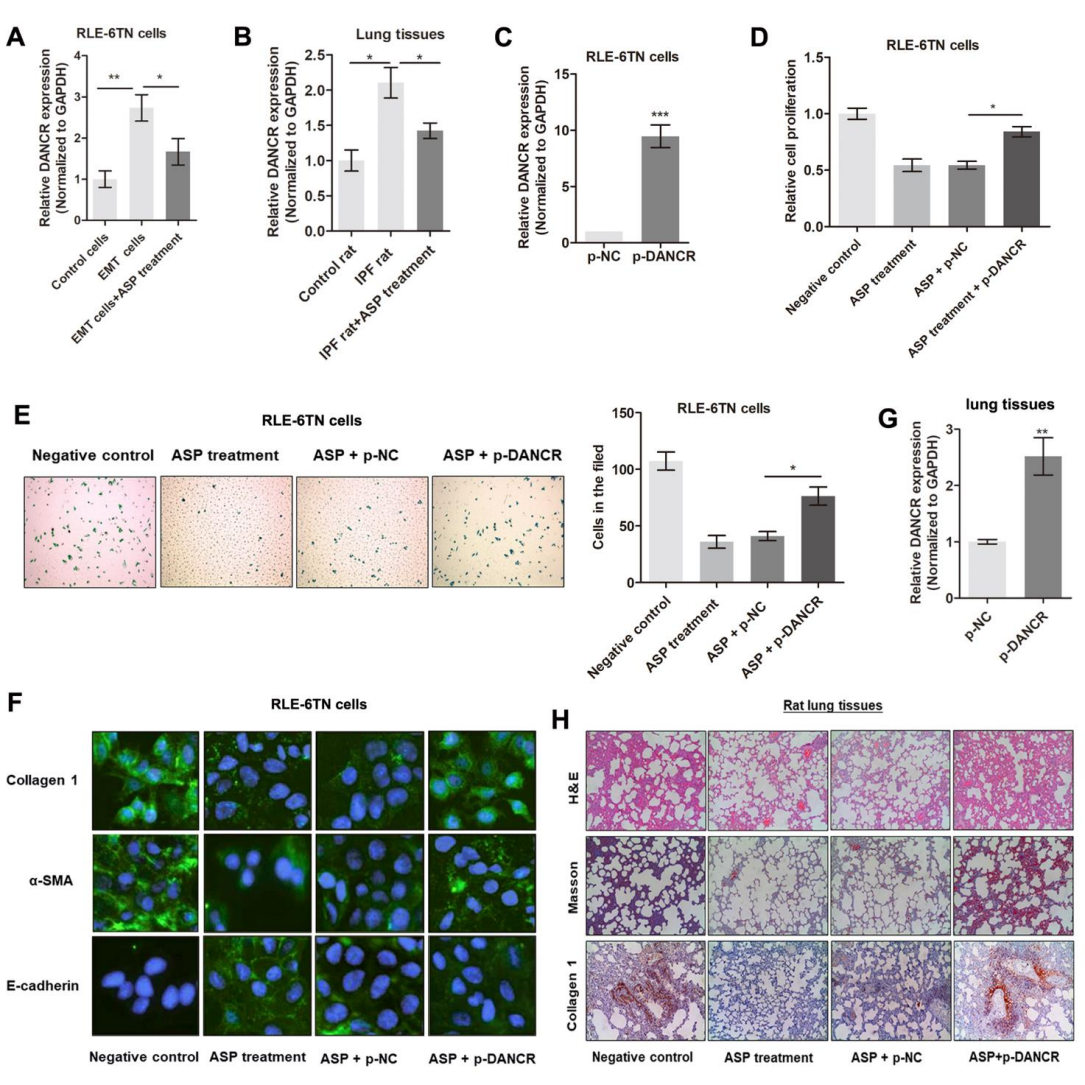
**LncRNA DANCR is essential for ASP-induced suppression of fibrogenesis**. **(A-B)** qRT-PCR was performed to determine the expression levels of DANCR in RLE-6TN cells (A) and rat lung tissues (B), which are treated with or without ASP, ^*^P<0.05, ^**^P<0.01. **(C)** DANCR was overexpressed in RLE-6TN cells by infection with DANCR-specific plasmid, ^***^P<0.001. **(D)** CCK8 assay revealed that DANCR overexpression attenuated ASP-induced suppression of cell proliferation, ^*^P<0.05. **(E)** Results of Transwell assay showed that ASP suppressed cell migration of RLE-6TN cells; however, transfection with DANCR reversed this effect, ^*^P<0.05. **(F)** Immunofluorescence assays showed that upregulation of DANCR levels abrogated the ASP-induced inhibition of collagen-1, E-cadherin, and α-SMA expression. Green fluorescence represents respective proteins (collagen-1, E-cadherin, and α-SMA), and blue fluorescence indicates nuclei stained by DAPI. **(G)** qPCR analysis was performed to evaluate the upregulation of DANCR levels in rats administered with p-DANCR compared to those in control rats, ^**^P<0.01. **(H)** H&E and Masson’s staining assays were performed to investigate the effects of DANCR in ASP-induced suppression of pulmonary fibrosis in rat lung tissues; immunohistochemical analysis of collagen-1 protein expression in rat lung tissues overexpressing DANCR.

### Western blots and antibodies

The total proteins were prepared, and their concentrations were determined using a Total Protein Extraction Kit (Solarbio, Beijing, China). Cell lysates were separated by 10% SDS-PAGE and transferred onto nitrocellulose membranes (GE Healthcare, USA). Then, the membranes were blocked with 5% non-fat milk in TBST buffer. The following primary antibodies were used in the study: anti-AUF1 (1:1000, Abcam, ab61193), anti-FOXO3 (1:1000, Abcam, ab17026), anti-E-cadherin (1:1000, Abcam, ab1416), anti-Collagen 1 (1:1000, Abcam, ab6308), anti-α-SMA (1:1000, Abcam, ab5694), and anti-β-actin (1:1000, Abcam, ab8226). Next, the membranes were incubated with HRP-conjugated secondary goat anti-mouse (1:5000, Proteintech, Rosemont, IL, SA00001-1) or goat anti-rabbit (1:5000, Proteintech, SA00001-2) antibodies for 2 h at room temperature. The relative grey values of immunoreactive bands were calculated relative to those of β-actin levels.

### Microarray analysis

Total RNA was extracted using the Trizol reagent and further purified using a Qiagen RNeasy Mini Kit according to the manufacturer’s instructions (Qiagen, Hilden, Germany). Microarrays were imaged on an Affymetrix GeneTitan® Multi-Channel Instrument. Each group was assayed on a single custom microarray. Microarrays were manufactured and processed in interconnected sets of 384. Next-generation sequencing data were produced on an Illumina HiSeq® 2500 (San Diego, Calif., USA) instrument according to the manufacturer’s instructions.

### Statistical analysis

Data were collected from at least three independent experiments and represented as mean ± standard deviation (SD). Data were analyzed using SPSS 19.0 statistical software. The statistical significance of quantitative assays was analyzed using either two-tailed Student’s *t*-test or ANOVA analysis for more than two groups. P-value < 0.05 was considered statistically significant. Data were presented as mean ± SD.

## RESULTS

### ASP treatment inhibits pulmonary fibrosis in vivo and cell fibrogenesis in vitro

To determine the role of ASP in pulmonary fibrosis, we first verified pulmonary fibrosis model in BLM-treated rats. Results of H&E and Masson’s trichrome staining assays showed higher collagen deposition in BLM-treated rats to those of the control rats (Fig. 1A). However, treatment with ASP significantly reversed BLM-induced collagen deposition. Results of IHC assay verified that BLM-treated rats had higher collagen-1 protein levels in lung tissues compared to those of rats treated with PBS; ASP treatment restored collagen-1 levels (Fig. 1A).

In addition, we examined effects of ASP on cell growth and EMT in RLE-6TN cells treated with TGF-β1. TGF-β1 treatment induced fibrogenesis, as evidenced by the disappearance of intercellular junctions and spindle-like structures (Fig. 1B). Results of CCK8 assay and Transwell assay verified the significant increase in growth rate and migratory ability of RLE-6TN cells cultured with TGF-β1, respectively; however, these effects were dramatically reversed by ASP treatment (Fig. 1C and 1D). In addition, ASP treatment abrogated the TGF-β1-induced upregulation of α-SMA and downregulation of E-cadherin expression in RLE-6TN cells (Fig. 1E).

### DANCR attenuates the suppressive effect of ASP on pulmonary fibrosis

To determine whether the lncRNA DANCR participates in mediating the effects of ASP in IPF, we measured DANCR expression levels in RLE-6TN cells and rat lung tissues. As shown in Figure 2A, DANCR levels were upregulated in TGF-β1-induced EMT cells compared to those in control cells; however, DANCR upregulation was dramatically reversed by ASP treatment. Moreover, we observed higher DANCR expression in IPF rats compared to control rats, and ASP treatment significantly reversed the upregulation of DANCR in IPF rats (Fig. 2B). We then generated adenovirus-carrying plasmid loading DANCR (p-DANCR) or a negative control (p-NC) in RLE-6TN cells treated with ASP (Fig. 2C). Results of CCK8 and Transwell migration assays showed that DANCR overexpression significantly abrogated ASP-induced suppressive effect on cell proliferation and migration (Fig. 2D-E). In addition, we investigated the effect of DANCR on fibrogenesis by measuring the expression levels of EMT-related protein markers. Immunofluorescence analysis showed that overexpression of DANCR restored the fibroblast activation of ASP-treated RLE-6TN cells, including upregulation of α-SMA and collagen 1 expression levels and the downregulation of E-cadherin expression (Fig. 2F).

To confirm the role of DANCR in ASP-induced IPF suppression, rats were administered p-DANCR or p-NC at the beginning of ASP treatment. DANCR expression levels were significantly upregulated in lung tissues after p-DANCR administration compared to those in p-NC control tissues (Fig. 2G). Results of H&E and Masson’s staining showed that ZEB1-AS1 upregulation significantly attenuated the ASP-mediated suppression of IPF (Fig. 2H). IHC analysis indicated that the downregulation of collagen-1 levels caused by ASP treatment was relieved in the lung tissues of p-DANCR-treated mice compared to those treated with p-NC (Fig. 2H). Our findings suggested that ASP suppresses IPF by downregulating DANCR expression.

**Figure 3.**
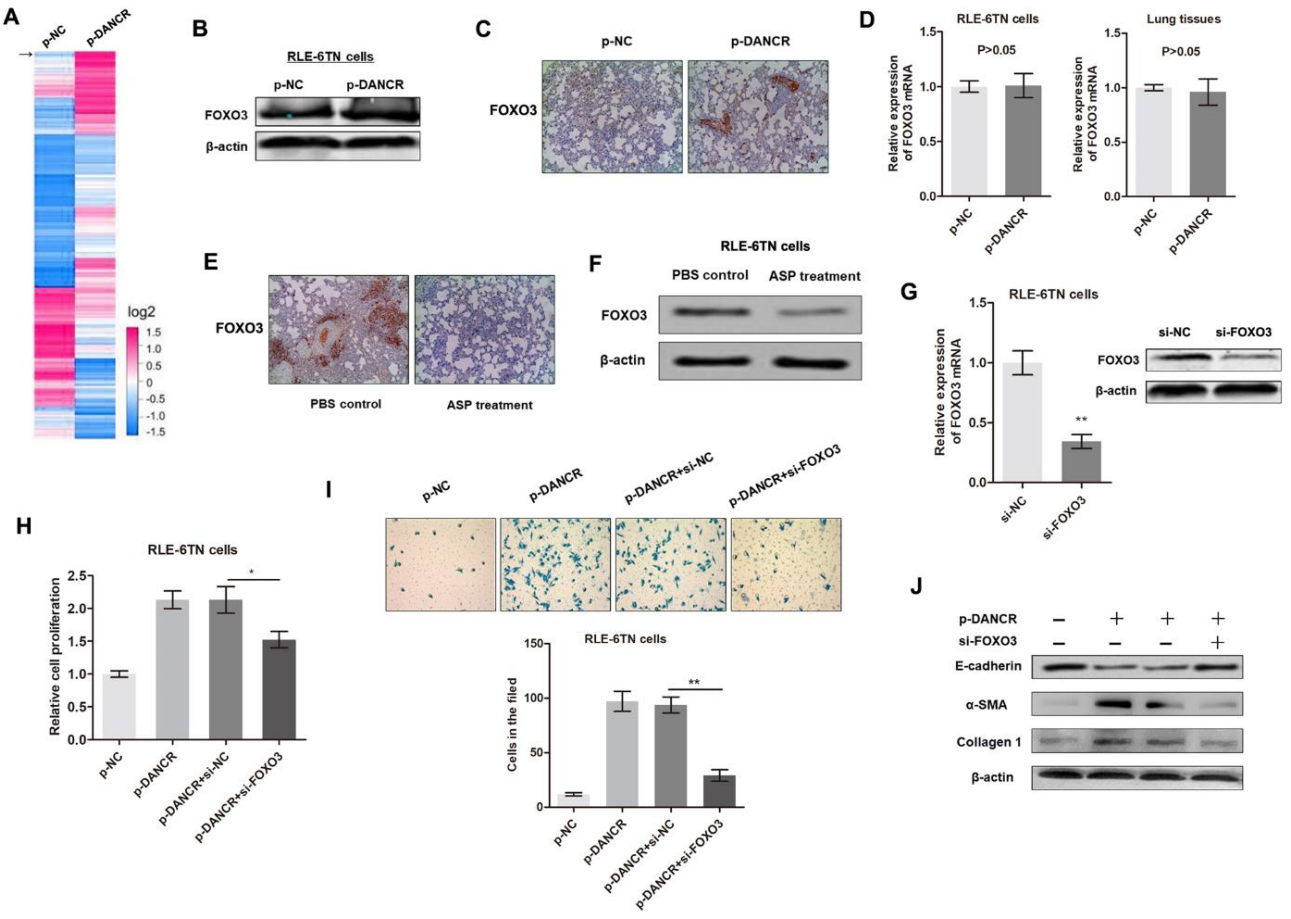
**DANCR regulates fibrogenesis by inducing FOXO3 expression**. **(A)** Heatmap showing mRNA expression levels in RLE-6TN cells transfected with control or DANCR plasmid for 48 h. Arrow indicates FOXO3. **(B)** FOXO3 expression was measured by western blot analysis in cells overexpressing DANCR. **(C)** FOXO3 protein levels in rat lung tissues were analyzed by immunohistochemistry. **(D)** FOXO3 mRNA expression was detected in RLE-6TN cells (left panel) and rat lung tissues (right panel) by qRT-PCR. **(E)** Immunohistochemical analysis of FOXO3 levels in lung tissues treated with ASP or PBS control. **(F)** Western blot assay was performed to detect FOXO3 protein levels in RLE-6TN cells treated with ASP or PBS control. **(G)** FOXO3 expression was silenced in RLE-6TN cells by transfection with FOXO3 siRNA, ^**^P<0.01. **(H)** Relative cell proliferation was measured by CCK8 assay in cells overexpressing DANCR and (or) FOXO3 knockdown cells, ^*^P<0.05. **(I)** Cell migration was evaluated by Transwell assay in cells overexpressing DANCR and (or) FOXO3 knockdown cells, ^**^P<0.01. **(J)** Effect of FOXO3 and DANCR on the expression of EMT-related proteins were identified by western blotting.

### DANCR regulates fibrogenesis via FOXO3 upregulation

To investigate the regulatory mechanism by which DANCR promotes the EMT and pulmonary fibrosis, we sought to identify the differentially expressed genes between DANCR-overexpressing cells and control cells. By performing a microarray analysis, we screened 815 differentially expressed mRNAs (fold change > 1.5) that are potential targets of DANCR (Fig. 3A). Several of these mRNAs, such as FOXO3, Snail1, and SMAD4, were demonstrated to be critical regulators of EMT and fibrogenesis [30]. Our preliminary findings indicated that Snail1 and SMAD4 have no functional interactions with DANCR. Therefore, we focused on FOXO3 to identify its functional role in DANCR-mediated lung fibroblasts. As expected, overexpression of DANCR increased the protein levels of FOXO3 in RLE-6TN cells (Fig. 3B). IHC analysis of the rat lung tissues showed that FOXO3 expression was upregulated in the DANCR-over-expression group relative to that in the control group (Fig. 3C). However, qRT-PCR analysis of FOXO3 mRNA levels showed that the influence of DANCR on FOXO3 mRNA expression was insignificant both *in vitro* and *in vivo* (Fig. 3D), which strongly suggested that DANCR regulates FOXO3 protein levels without influencing its mRNA levels. Moreover, ASP treatment dramatically suppressed FOXO3 protein expression *in vitro* and *in vivo* (Fig. 3E and 3F), which is consistent with the observed interaction between DANCR and FOXO3.

Then, we evaluated the functional role of FOXO3 in DANCR-mediated EMT and fibrogenesis in RLE-6TN cells by DANCR overexpression and FOXO3 knockdown (Fig. 3G). As shown in Figure 3H-I, upregulation of DANCR promoted RLE-6TNcell proliferation and migration; however, this effect was significantly reversed by FOXO3 knockdown. In addition, western blot analysis showed that FOXO3 knockdown abrogated the effect of DANCR on EMT in RLE-6TN cells (Fig. 3J). The above findings demonstrated that DANCR regulates EMT and fibrogenesis by upregulating FOXO3 protein levels without influencing mRNA expression.

**Figure 4.**
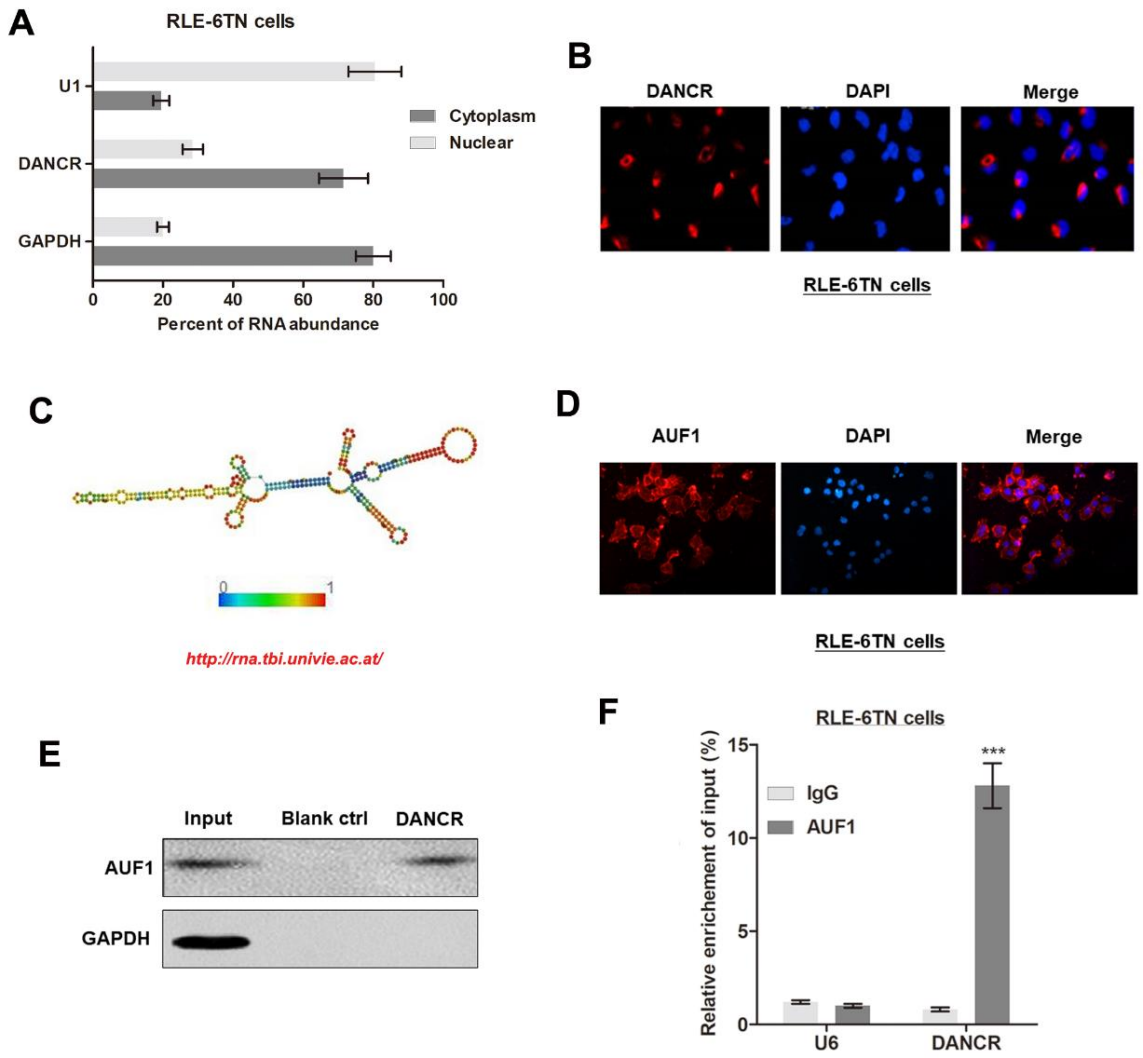
**LncRNA DANCR is associated with AUF1 in RLE-6TN cells**. **(A)** Nuclear fraction experiments and qRT-PCR experiments were performed to determine the relative distribution of DANCR in nucleus and cytoplasm of RLE-6TN cells. **(B)** The distribution of DANCR was determined by performing RNA fluorescence in situ hybridization (FISH) in RLE-6TN cells. **(C)** Prediction of 350-670-nt DANCR structures based on minimum free energy (MFE) and partition function (http://rna.tbi.univie.ac.at/). **(D)** Immunofluorescence assay was performed to identify the subcellular distribution of AUF1 proteins in RLE-6TN cells. **(E)** RNA pulldown followed by western blotting was performed with DANCR probe to verify the direct interaction between AUF1 and DANCR. GAPDH was used as the internal control. **(F)** RNA immunoprecipitation (RIP) was performed with an anti-AUF1 antibody to identify the association between DANCR and AUF1. Enrichment was shown as the percentage of input, ^***^P<0.001.

**Figure 5.**
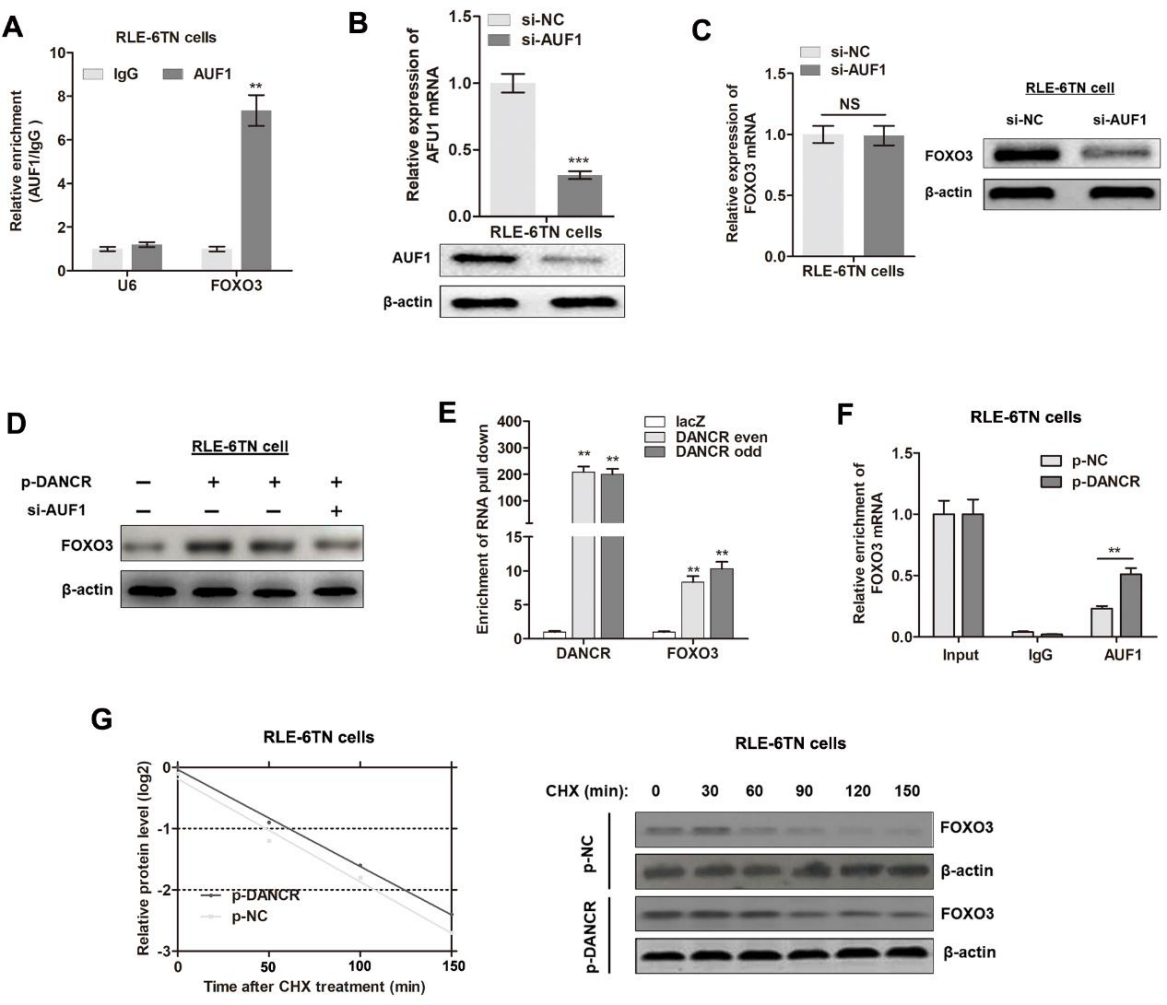
**DANCR promotes translation of FOXO3 gene by binding to AUF1**. **(A)** RIP was performed using anti-AUF1 and control IgG antibodies, followed by qRT-PCR to determine the enrichment of DANCR and U6. U6 was used as the negative control, ^**^P<0.01. **(B)** Experimental validation of AUF1 knockdown in RLE-6TN cells by qRT-PCR and western blotting, ^***^P<0.001. **(C)** mRNA and protein levels of FOXO3 in RLE-6TN cells silenced with AUF1. **(D)** Western blotting was performed to detect the protein expression levels of FOXO3 in RLE-6TN cells overexpressing DANCR or (and) silenced with AUF1. **(E)** RLE-6TN lysates were incubated with *in vitro*-synthesized, biotin-labeled control LacZ DNA probes or DNA probes against DANCR for the biotinylated oligonucleotide pulldown assay. ^**^P<0.01 compared to respective LacZ probes. **(F)** Endogenous AUF1 binding to FOXO3 mRNA was modified by DANCR overexpression, ^**^P<0.01. **(G)** Control plasmids or DANCR plasmids transfected RLE-6TN cells were cultured with 20 μg/ml cycloheximide (CHX) for 0-150 min and analyzed by western blotting.

### LncRNA DANCR is associated with AUF1

To identify the subcellular location of DANCR in alveolar epithelial cells, we performed a serious of experimental assays, including cellular fractionation and RNA-FISH. Both assays revealed that DANCR was primarily located in the cytoplasm (Fig. 4A and B), which suggested that DANCR can regulate downstream signaling at the post-transcriptional level [21, 31]. To investigate the underlying mechanism by which DANCR regulates FOXO3, we analyzed the secondary structure of DANCR. Based on minimum free energy (MFE) and partition function (http://rna.tbi.univie.ac.at/), we predicted that DANCR transcript at the 350-670 nt loci formed a stem-loop structure (Fig. 4C), which is critical for physical interactions with proteins. To verify the proteins associated with DANCR in RLE-6TN cells, RNA pulldown assay was performed, followed by mass spectrometry. The analysis identified a list of potential DANCR-interacting proteins (Table 2), including AU-binding factor 1 (AUF1). AUF1 could bind to (A + U)-rich elements (AREs) located within the 3ʹ untranslated regions (UTRs) of target mRNAs and promote translation without affecting the mRNA levels [32]. We determined the subcellular localization of AUF1 in RLE-6TN cells by conducting immunofluorescence assay, and results revealed that AUF1 is strongly expressed in the cytoplasm (Fig. 4D). Moreover, RNA pull-down assay showed that AUF1 proteins were significantly precipitated by a specific DANCR probe (Fig. 4E). Results of RIP assay verified that DANCR was pulled down by AUF antibody (Fig. 4F). Collectively, our results suggested that DANCR could interact with AUF1 in RLE-6TN cells.

**Figure 6.**
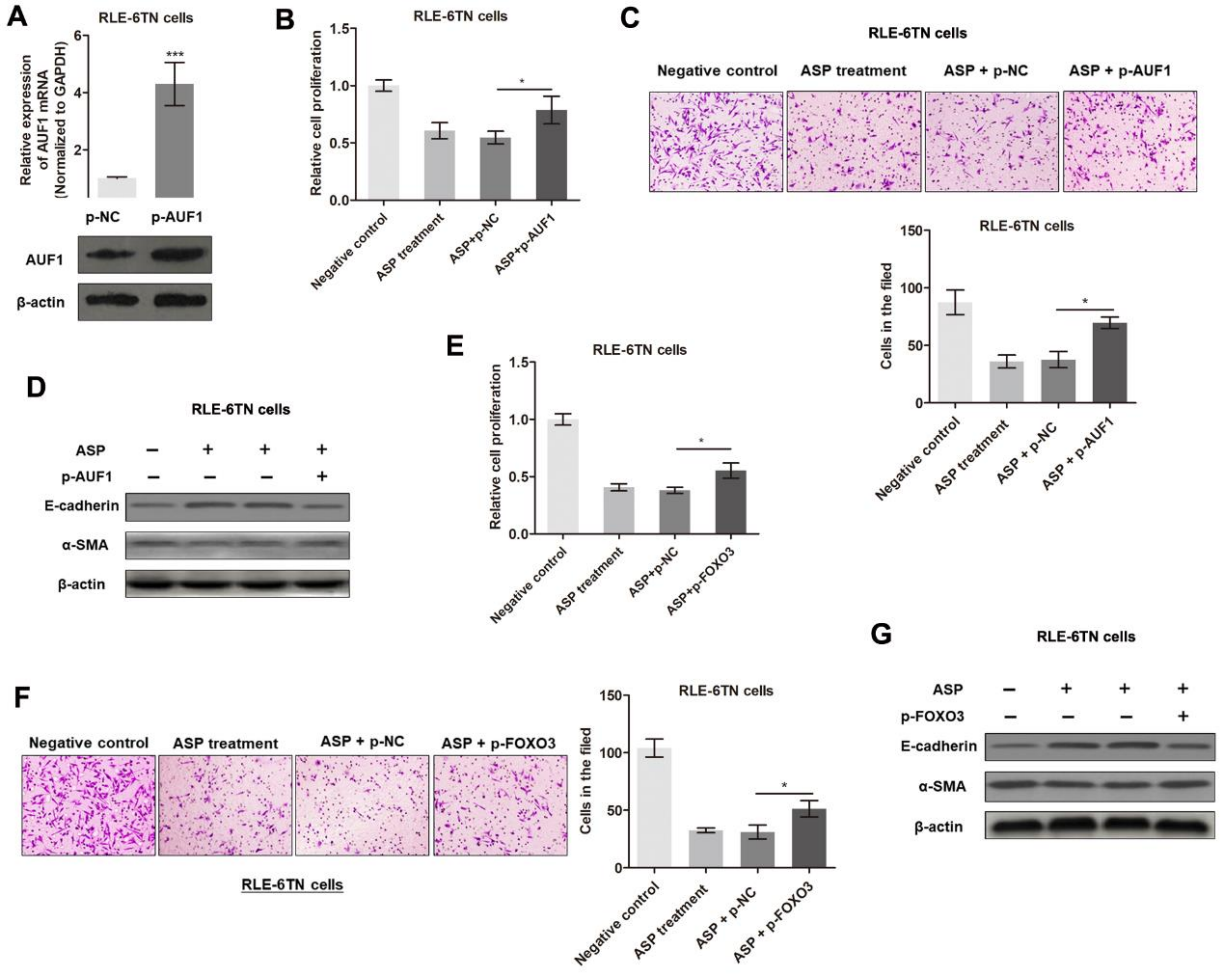
**ASP suppresses fibrogenesis by regulating the DANCR/AUF1/FOXO3 axis**. **(A)** AUF1 overexpression was validated by qRT-PCR and western blotting, ^***^P<0.001. **(B)** Cell proliferation was measured by CCK8 assay in cells treated with ASP and (or) p-AUF1, ^*^P<0.05. **(C)** Cell migration was evaluated by Transwell assay in cells treated with ASP and(or) p-AUF1, ^*^P<0.05. **(D)** Expression of EMT-relevant proteins, E-cadherin, and α-SMA were detected by western blot experiments in cells treated with ASP and (or) AUF1 plasmid. **(E)** Cell proliferation was measured by CCK8 assay in cells treated with ASP and (or) p-AUF1, ^*^P<0.05. **(F)** Cell migration was evaluated by Transwell assay in cells treated with ASP and (or) p-AUF1, ^*^P<0.05. **(G)** Expression levels of EMT-relevant proteins, E-cadherin, and α-SMA, were detected by western blot experiment in cells treated with ASP and (or) AUF1 plasmid.

### LncRNA DANCR activates the translation of FOXO3 mRNA by recruiting AUF1

Based on the above results, we hypothesized that DANCR promoted FOXO3 mRNA translation by binding to AUF1. To test this hypothesis, we performed RIP assay and results showed that FOXO3 was enriched in AUF1 precipitates (Fig. 5A). Knockdown of AUF1 (Fig. 5B) led to the downregulation of FOXO3 protein levels without affecting FOXO3 mRNA levels (Fig. 5C). Moreover, silence of AUF1 reversed DANCR-induced upregulation of FOXO3 protein (Fig. 5D). Considering that DANCR and AUF1 synergistically regulate FOXO3 expression, we reasoned that DANCR can upregulate FOXO3 protein levels by recruiting AUF1 to bind to the FOXO3 3ʹ-UTR. In support of this hypothesis, endogenous FOXO3 mRNA was co-precipitated with DANCR in RLE-6TN cells by biotinylated oligonucleotide pulldown assay (Fig. 5E), and over-expressed DANCR promoted endogenous AUF1 binding to FOXO3 mRNA by RIP (Fig. 5F). In addition, RLE-6TN cells were treated with cycloheximide (CHX), which inhibited the active synthesis and secretion of proteins. We measured the degradation of existing proteins, and results revealed that dysregulated DANCR levels did not affect the half-life of FOXO3 proteins in RLE-6RN cells (Fig. 5G), indicating that DANCR did not affect FOXO3 protein degradation. Taken together, our results demonstrated that DANCR guides the binding of AUF1 to FOXO3 mRNA, thereby activating its translation without affecting mRNA levels.

**Table 2:** Identification of DANCR binding proteins by MS.

Protein	Beads	DANCR	Ratio (DANCR/Beads)
AUF1	0	3	NA
STT3B	0	3	NA
ARF6	0	3	NA
DPM1	1	3	3
SMAD4	0	3	NA
LAS1L	1	3	3
AKAP8	0	3	NA
GSTO1	0	3	NA
ZEB2	0	3	NA
PCH2	0	3	NA

MS: mass spectrometry; Beads: spectral counts of proteins in beads only group; DANCR: spectral counts of proteins in DANCR group; Ratio (DANCR/Beads): spectral count ratio of proteins comparing DANCR group to beads only group; NA: not available.

### ASP modulates the EMT and IPF via the DANCR/AUF1/FOXO3 regulatory axis

After having validated the direct interactions among DANCR, AUF1, and FOXO3, we sought to verify whether APS mediates IPF by inhibiting the DANCR/AUF1/FOXO3 pathway. AUF1 was overexpressed in RLE-6TN cells with specific plasmids (Fig. 6A). Enhanced AUF1 reversed the ASP-mediated suppression of cell proliferation, migration, and the EMT (Fig. 6B-D). In addition, FOXO3 overexpression restored the inactivation of fibrogenesis induced by APS (Fig. 6E-G). Taken together, our findings demonstrated that ASP suppresses EMT and lung fibrogenesis by suppressing the DANCR/AUF1/FOXO3 signaling pathway.

**Figure 7.**
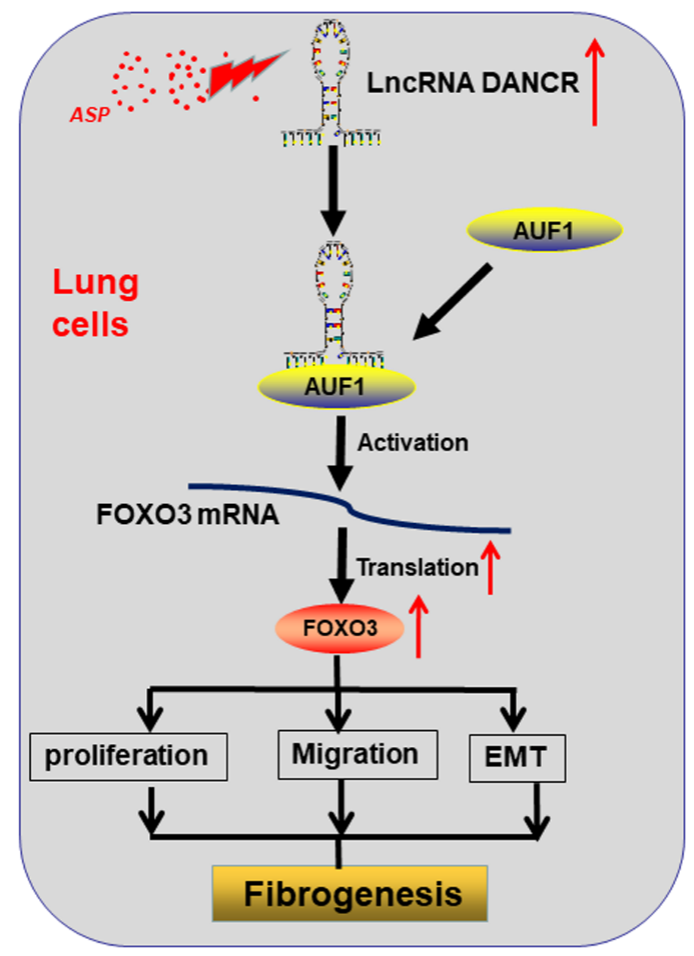
**Schematic showing the proposed regulatory mechanisms of ASP**. ASP treatment upregulated DANCR expression. DANCR overexpression upregulates FOXO3 expression by guiding AUF1 to activate the translation of FOXO3 mRNA, thereby promoting proliferation, migration, and the EMT and initiation of pulmonary fibrosis.

Overall, our study findings showed that ASP suppresses IPF by downregulating DANCR expression, which post-transcriptionally inactivates FOXO3 translation in an AUF1-dependent manner (Fig. 7). Therefore, ASP could be used for the treatment of IPF, and DANCR is a promising therapeutic target for significantly improving treatment efficiency for IPF patients.

## DISCUSSION

IPF is an aggressive end-stage disease, and current therapeutic treatments for IPF patients are currently very limited. As a result, many patients turn to alternative treatment [33]. Recent years have witnessed an increasing interest in herbal remedies in the industrialized world, since these drugs are increasingly considered as effective and safe alternatives to synthetic drugs. In the present study, we investigated ASP, one of the few well-established plant products extracted from the roots of angelica. Our results indicated that ASP could prevent IPF progression both *in vitro* and *in vivo*. Moreover, ASP downregulates the expression of lncRNA DANCR, thereby leading to post-transcriptional inactivation of FOXO3 translation in an AUF1-dependent manner.

Accumulating evidence showed that ASP is useful for the treatment of various diseases, including cancers and ulcers, because of its anti-oxidative, anti-proliferative, and immunomodulatory effects [4]. For example, Liao et al. demonstrated that ASP could prolong the survival rates of gastric cancer patients and cause cell death in gastric cancer cells by activating the intrinsic mitochondrial pathway [34]; Ye et al. showed that ASP promoted gastric ulcer healing and reduced ulcer area of gastric [35]. Importantly, one study by Wang et al. suggested that ASP was effective in treating and alleviating interstitial pulmonary fibrosis in rats, possibly by lowering collagen levels, inhibiting NF-κB activity, and downregulating TGF-β expression [36]. Our study findings were consistent with the results reported by Wang et al. and demonstrated that ASP inhibits IPF progression both *in vitro* and *in vivo*, suggesting that ASP may be useful for the treatment of IPF in humans.

The mechanism by which ASP exerts its protective effects in IPF remains unclear. To identify the pathways regulated by ASP in IPF, we focused on lncRNAs that are involved in the regulation of almost every stage of gene expression and are implicated in various disease states via multiple regulatory mechanisms [37]. Multiple studies suggested that DANCR could act as an oncogene by competing with other microRNAs, such as miR-496, miR-1972, and miR-577 [21, 38, 39]. DANCR was found to promote cancer initiation and progression in various cancer types, including lung cancer and osteosarcoma. Importantly, several studies indicated that DANCR could play a critical roles in the EMT and migration of cancer cells [40] and suggested that DANCR participates in the initiation and progression of IPF. As expected, our results showed that DANCR levels were downregulated by ASP treatment. More importantly, DANCR upregulation reversed the inhibitory effects of ASP on IPF progression. Herein, our study is the first to show that DANCR is essential for the suppressive effects of ASP on IPF.

We aimed to determine the target genes of DANCR. Genome-wide mRNA expression profiling was performed to identify the differentially expressed mRNAs. One of these mRNAs was FOXO3, which we previously verified as a critical regulator of TGF-β1-induced EMT and fibrogenesis [27], thereby suggesting that FOXO3 is linked to EMT and fibrogenesis. Consistent with the notion that FOXO3 is a critical downstream integrator of DANCR and TGF-β1, FOXO3 knockdown abrogated the effects of DANCR in TGF-β1-induced EMT and fibrogenesis. LncRNAs that are located in the nucleus or cytoplasm can have different functional roles [41]. Results showed that DANCR is primarily distributed in the cytoplasm of RLE-6TN cells, indicating that it can regulate IPF at the post-transcriptional level. The gain-or-loss function assay showed that DANCR upregulates FOXO3 protein expression without affecting its mRNA levels, thereby providing further evidence that this functional interaction occurs at the post-transcriptional level.

To unravel the mechanisms by which DANCR regulates FOXO3 protein levels, screening identified AUF1 as a DANCR-interacting protein. AUF1 is a protein family comprising four RNA-binding proteins (RBPs) generated by alternative pre-messenger RNA (pre-mRNA) splicing that plays canonical roles in controlling the stability or translation of mRNA targets based on recognition of AU-rich sequences within the 3'-UTRs of their target mRNA [42]. Our results validated that AUF1 associates with DANCR and can act as an adaptor protein that cooperates with DANCR to bind to FOXO3 mRNA. Given that DANCR influenced FOXO3 protein levels without affecting the mRNA levels, we assumed that AUF1 regulates the translation of FOXO3 mRNA. Our RIP results verified the direct interaction between AUF1 and FOXO3. Moreover, DANCR did not affect FOXO3 protein stability, which confirmed our assumption.

Recent studies revealed the therapeutic potential of lncRNAs by designing specific silencing molecules to knock off the specific oncogenic lncRNAs in cancer [43]. Additionally, siRNA or locked nucleic acids can serve as therapeutic agents that specifically target lncRNAs *in vivo* [44]. Therefore, inhibition of IPF progression via ASP combined with small molecules that improve ASP treatment efficiency are potential therapeutic methods. The present study has certain limitations. Although we performed *in vivo* studies confirming the essential role of DANCR in ASP-induced suppression of IPF progression, a more comprehensive *in vivo* validation of the *in vitro* results involving the functional regulation of DANCR/AUF1/FOXO3 axis is required. Future studies should be conducted to strengthen our findings.

In summary, our findings showed that ASP could downregulate the expression of DANCR, which in turn represses AUF1-mediated FOXO3 translation to suppress the EMT and pulmonary fibrosis. Identifying the detailed functional mechanisms of ASP/DANCR/FOXO3 regulatory axis in the initiation of IPF will improve our understanding of the therapeutic effects of herbal medicine in IPF and contribute to the development of novel anti-IPF strategies.
